# Shifts in dental trauma research (2000–2025): a literature-based thematic analysis using topic modeling

**DOI:** 10.1186/s12903-026-08446-9

**Published:** 2026-04-29

**Authors:** Mustafa Taha Guller, Hatice Guller, Burkay Moroğlu, Ruşen Erdem, Ahmet Yıldırım, Yavuz Selim Genç

**Affiliations:** 1 Department of Oral and Dentomaxillofacial Radiology, Faculty of Dentistry, Giresun University, Giresun, 28100 Türkiye; 2 Department of Orthodontics, Faculty of Dentistry, Atatürk University, Erzurum, Türkiye; 3 Department of Orthodontics, Faculty of Dentistry, Kafkas University, Kars, Türkiye; 4 Department of Orthodontics, Faculty of Dentistry, Bulent Ecevit University, Zonguldak, Türkiye; 5 Department of Orthodontics, Faculty of Dentistry, Giresun University, Giresun, Türkiye

**Keywords:** Bertopic, Dental injuries, Dental trauma, Natural language processing, Topic modeling

## Abstract

**Objectives:**

The aim of this study is to analyze the structural, conceptual, and temporal evolution of the dental trauma literature using the BERTopic algorithm, which can identify latent semantic patterns in large-scale textual data, and to reveal trends based on publication year, country, and journal.

**Materials and methods:**

The keywords “dental trauma*” and “dental injur*” were searched in the title, abstract, and keyword fields of the Scopus database. Only English-language journal articles and conference papers published between 2000 and 2025 were included. Records lacking abstracts, institutional affiliations, or source titles were excluded. After applying these criteria, a total of 3,473 records were analyzed. BERTopic analysis was performed using transformer-based embedding, dimensionality reduction, and density-based clustering techniques.

**Results:**

Thirteen themes reflecting the multifactorial structure of the dental trauma literature were identified. The theme “Dental Trauma and Oral Health-Related Quality of Life” represented the hottest topic, whereas “Dental Fractures in Traumatic Injuries” was identified as the coldest topic. A marked increase in publication volume was observed after 2006, and Brazil ranked first in terms of publication output.

**Conclusions:**

Dental trauma research has long been clinically oriented but has recently evolved into a multifaceted field encompassing biological, educational, behavioral, and engineering aspects. However, technological approaches such as artificial intelligence have yet to gain presence in the literature.

**Clinical relevance:**

To the best of our knowledge, this study provides the first comprehensive mapping of the thematic orientation, temporal trends, and conceptual evolution of the dental trauma literature. It offers a clear overview of how dental trauma research has evolved over the past 25 years, enabling clinicians to understand shifts in clinical priorities and align their practices with current approaches.

## Introduction

Traumatic dental injuries (TDI) occur as a result of sudden and unexpected external forces acting on the teeth and surrounding tissues, and they are mostly caused by accidents [1]. Rather than being a pathological process, these injuries arise from the unpredictable circumstances of life and can significantly affect both the individual’s oral functions and overall quality of life. Children, adolescents, and individuals participating in sports activities are particularly at risk; however, people of all age groups are potentially susceptible [2]. Epidemiological studies conducted on a global scale have revealed that the annual incidence of TDI is approximately 4.5%, while the prevalence ranges between 6% and 59% [3].

In recent years, the increasing volume of publications has rapidly expanded the knowledge base in the field of dental trauma; however, it has also made it challenging to evaluate the existing literature in a comprehensive and systematic manner. At this point, natural language processing (NLP)–based approaches offer researchers significant advantages. Although traditional topic modeling methods have been used in text mining for many years, they may be limited in producing meaningful and interpretable themes within large datasets [4].

In parallel with the growing body of literature, several approaches have been employed to synthesize and interpret trends in dental trauma research. Evidence mapping studies have primarily focused on categorizing existing evidence according to predefined clinical domains and assessing the methodological quality of systematic reviews or randomized controlled trials, thereby identifying areas with strong or weak evidence bases [5–7]. Similarly, bibliometric analyses have been widely used to describe publication outputs, citation patterns, influential authors, journals, and countries contributing to the field [8]. While these approaches provide valuable insights into the distribution, volume, and quality of research, they are generally limited in capturing the latent thematic structure of the literature and its temporal evolution based on the semantic content of publications. In particular, both evidence mapping and bibliometric analyses rely on predefined categories, metadata, or citation-based indicators, which may not fully reflect how the conceptual focus of dental trauma research has shifted over time.

To address these limitations, BERTopic is built upon Google’s BERT (Bidirectional Encoder Representations from Transformers) language model and was developed by Maarten Grootendorst [9]. This algorithm groups large volumes of textual data (such as articles, theses, or reports) according to their content similarities, thereby revealing structural patterns within the field [10]. Unlike traditional methods, it does not merely count word frequencies but also considers the semantic relationships of words within their contextual usage [11].

The aim of this study is to thematically examine the dental trauma literature using the BERTopic algorithm and to make the structural, conceptual, and temporal dynamics of development within the field more visible. This method systematically identifies the dominant themes of dental trauma, emerging research areas, and less-explored topics by clustering studies in the literature according to their semantic similarities. The findings contribute both to defining future research priorities for investigators and to clarifying the areas of knowledge concentration and gaps within the existing literature. In this respect, by applying the BERTopic method to the field of dental trauma for the first time, this study introduces both a methodological and strategic innovation to the literature.

## Materials and methods

### Data source and literature review

The scientific literature on dental trauma was systematically analyzed using the BERTopic algorithm. Data were obtained from the Scopus database through a search conducted on August 12, 2025. This study was not designed as a systematic review or evidence mapping exercise, but rather to identify dominant thematic patterns and temporal trends in the dental trauma literature using large-scale topic modeling. Accordingly, the Scopus database was selected to ensure standardized metadata and analytical consistency for BERTopic analysis. The search was performed using the keywords “dental trauma*” and “dental injur*,” covering the title, abstract, and keyword fields. Retrieved records were exported using the Scopus export function. After excluding other publication types and considering only journal articles and conference papers, non-English studies were removed [12]. Entries lacking abstracts, institutional affiliations, or source titles were subsequently excluded. Duplicate records were also checked, and no repetitions were found.

### Data preprocessing

The collected records were subjected to a preprocessing phase to improve data quality. During the analysis, the available titles, abstracts, and author keywords were merged into a single column. For records lacking author keywords, only the title and abstract sections were included in the evaluation. In addition, common stop words with low semantic contribution (e.g., “the,” “and,” “or,” “of”) were removed from the text using the default list of the Natural Language Toolkit (NLTK) library. A domain-specific list containing frequently used but contextually limited terms (e.g., “trauma”) was also added. All these steps were applied to facilitate the extraction of more distinctive and meaningful topic clusters.

### BERTopic modeling process

To identify the main themes and latent structures within the dataset, the BERTopic algorithm, which relies on NLP techniques for topic modeling, was employed [9, 13, 14].

Titles and abstracts were converted into high-dimensional vectors using the S-PubMedBERT-MS-MARCO architecture, which is optimized for medical text processing [15, 16]. In the generated vector space, semantically related texts were positioned closer to each other [17]. Dimensionality reduction was performed using the Uniform Manifold Approximation and Projection (UMAP) technique [18]. Following this step, Hierarchical Density-Based Spatial Clustering of Applications with Noise (HDBSCAN), a density-based clustering approach, was applied [19]. The texts were tokenized into words and word groups, after which frequency-based matrices were created using the CountVectorizer tool [12].

To identify the distinguishing terms of each cluster, the Class-based Term Frequency–Inverse Document Frequency (c-TF–IDF) method was applied [9]. Using this approach, the most informative keywords of the clusters were determined. The parameters (n_neighbors = 15, min_cluster_size = 30) were optimized for consistency; higher n_neighbors values emphasized global trends, whereas lower values highlighted local relationships [18]. The min_cluster_size parameter determines whether the clusters are more general or more specific. Lower values generate smaller and more detailed clusters, which may also increase noise [20].

For each topic, representative keywords and related publications were reviewed by three experts with at least five years of experience in the field, and meaningful labels were assigned through consensus reached in group discussions [21]. To examine the temporal trends among topics, linear regression models were applied; topics showing a positive trend were categorized as “hot” topics, whereas those showing a negative trend were categorized as “cold” topics. In this way, the directional shifts in research focus over time were identified [22]. For each topic, Ordinary Least Squares (OLS) regression was applied to estimate the relationship between the annual topic proportion and the publication year, and the slope coefficient (β₁) was calculated. Subsequently, using the outputs of topic modeling, the dynamics of the research field were evaluated through more detailed analyses, including topic trends and distributions by countries and journals. Methodological decisions, including parameter selection and topic labeling, were guided by established BERTopic and NLP literature and were further evaluated through expert consensus.

During the data preprocessing stage, the Pandas and NumPy libraries were utilized, while the NLTK library was employed for the NLP phase. Topic modeling was conducted using the Sentence-Transformers and BERTopic libraries. Visualizations were generated with the WordCloud package. All computations and analyses in this study were performed in Python (version 3.13.5) on Visual Studio Code (version 1.101.1).

## Results

**Fig. 1:**
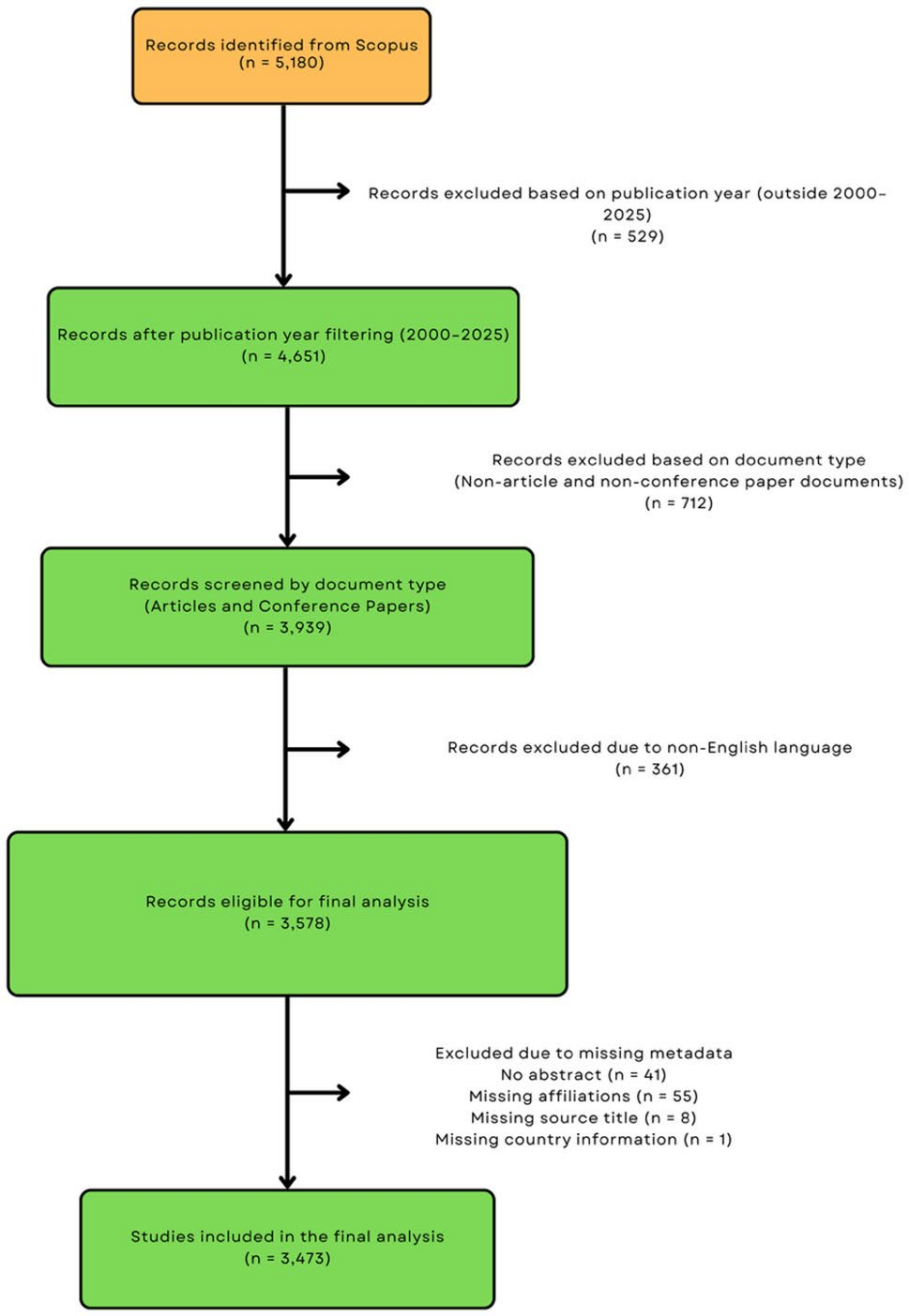
Flowchart illustrating the dataset construction process

The outcomes of the dataset construction process are presented in Fig. 1. The initial Scopus search identified 5,180 records. Sequential filtering based on publication year, document type, and language resulted in 3,578 records eligible for further evaluation. Subsequent screening for data completeness led to the exclusion of records with missing abstracts, affiliations, source titles, or country information. Following these steps, a total of 3,473 publications were included in the final BERTopic analysis.

**Fig. 2:**
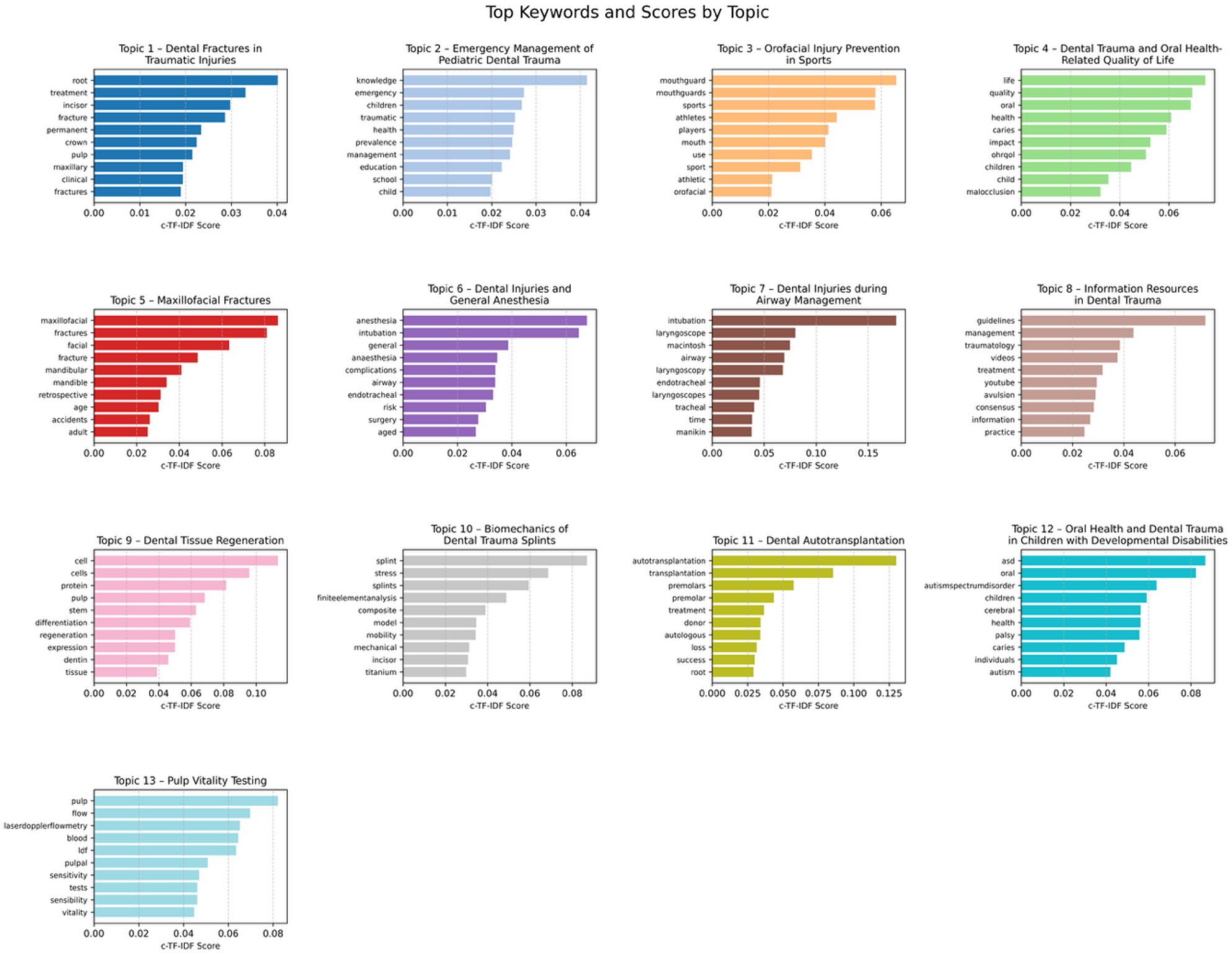
Top keywords and their corresponding scores by topic

By applying the BERTopic model, 13 distinct topics and their corresponding topic–word distributions were identified within the field of dental trauma. The keywords defining each topic and their respective c-TF–IDF scores are presented in Fig. 2. Table 1 summarizes all generated topics along with their representative keywords and the number of related publications. Each topic is characterized by a unique set of keywords that reflects the thematic structure of dental trauma research.

**Table 1:** Relevant keywords and publication counts for 13 distinct topics identified by the BERTopic model

Topic	Name	Representation	Count
T1	Dental Fractures in Traumatic Injuries	‘root’, ‘treatment’, ‘incisor’, ‘fracture’, ‘permanent’, ‘crown’, ‘pulp’, ‘maxillary’, ‘clinical’, ‘fractures’	881
T2	Emergency Management of Pediatric Dental Trauma	‘knowledge’, ‘emergency’, ‘children’, ‘traumatic’, ‘health’, ‘prevalence’, ‘management’, ‘education’, ‘school’, ‘child’	792
T3	Orofacial Injury Prevention in Sports	‘mouthguard’, ‘mouthguards’, ‘sports’, ‘athletes’, ‘players’, ‘mouth’, ‘use’, ‘sport’, ‘athletic’, ‘orofacial’	230
T4	Dental Trauma and Oral Health-Related Quality of Life	‘life’, ‘quality’, ‘oral’, ‘health’, ‘caries’, ‘impact’, ‘ohrqol’, ‘children’, ‘child’, ‘malocclusion’	206
T5	Maxillofacial Fractures	‘maxillofacial’, ‘fractures’, ‘facial’, ‘fracture’, ‘mandibular’, ‘mandible’, ‘retrospective’, ‘age’, ‘accidents’, ‘adult’	84
T6	Dental Injuries and General Anesthesia	‘anesthesia’, ‘intubation’, ‘general’, ‘anaesthesia’, ‘complications’, ‘airway’, ‘endotracheal’, ‘risk’, ‘surgery’, ‘aged’	83
T7	Dental Injuries during Airway Management	‘intubation’, ‘laryngoscope’, ‘macintosh’, ‘airway’, ‘laryngoscopy’, ‘endotracheal’, ‘laryngoscopes’, ‘tracheal’, ‘time’, ‘manikin’	79
T8	Information Resources in Dental Trauma	‘guidelines’, ‘management’, ‘traumatology’, ‘videos’, ‘treatment’, ‘youtube’, ‘avulsion’, ‘consensus’, ‘information’, ‘practice’	59
T9	Dental Tissue Regeneration	‘cell’, ‘cells’, ‘protein’, ‘pulp’, ‘stem’, ‘differentiation’, ‘regeneration’, ‘expression’, ‘dentin’, ‘tissue’	49
T10	Biomechanics of Dental Trauma Splints	‘splint’, ‘stress’, ‘splints’, ‘finiteelementanalysis’, ‘composite’, ‘model’, ‘mobility’, ‘mechanical’, ‘incisor’, ‘titanium’	48
T11	Dental Autotransplantation	‘autotransplantation’, ‘transplantation’, ‘premolars’, ‘premolar’, ‘treatment’, ‘donor’, ‘autologous’, ‘loss’, ‘success’, ‘root’	33
T12	Oral Health and Dental Trauma in Children with Developmental Disabilities	‘asd’, ‘oral’, ‘autismspectrumdisorder’, ‘children’, ‘cerebral’, ‘health’, ‘palsy’, ‘caries’, ‘individuals’, ‘autism’	32
T13	Pulp Vitality Testing	‘pulp’, ‘flow’, ‘laserdopplerflowmetry’, ‘blood’, ‘ldf’, ‘pulpal’, ‘sensitivity’, ‘tests’, ‘sensibility’, ‘vitality’	31

**Fig. 3:**
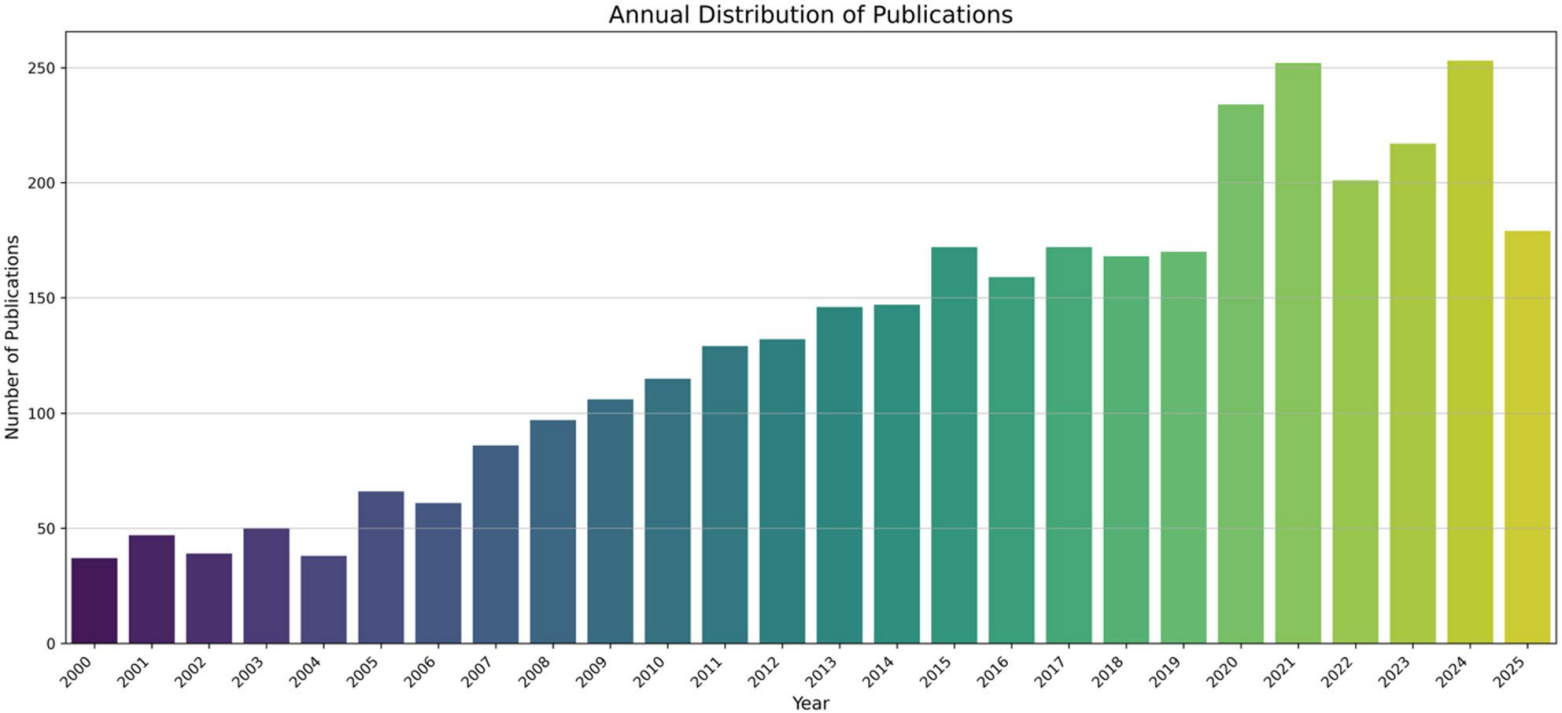
Annual distribution of publication counts

The distribution of publications in the field of dental trauma by year is presented in Fig. 3. While the number of publications was relatively low in the early 2000s, a gradual increase was observed after 2006. A notable rise in publications occurred starting from 2020. Although a slight decline was observed in 2022, an increase was again recorded in 2023 and 2024, with 2024 representing a new peak. The decrease observed in 2025 can be attributed to the fact that the year had not yet concluded at the time of data collection.

**Fig. 4:**
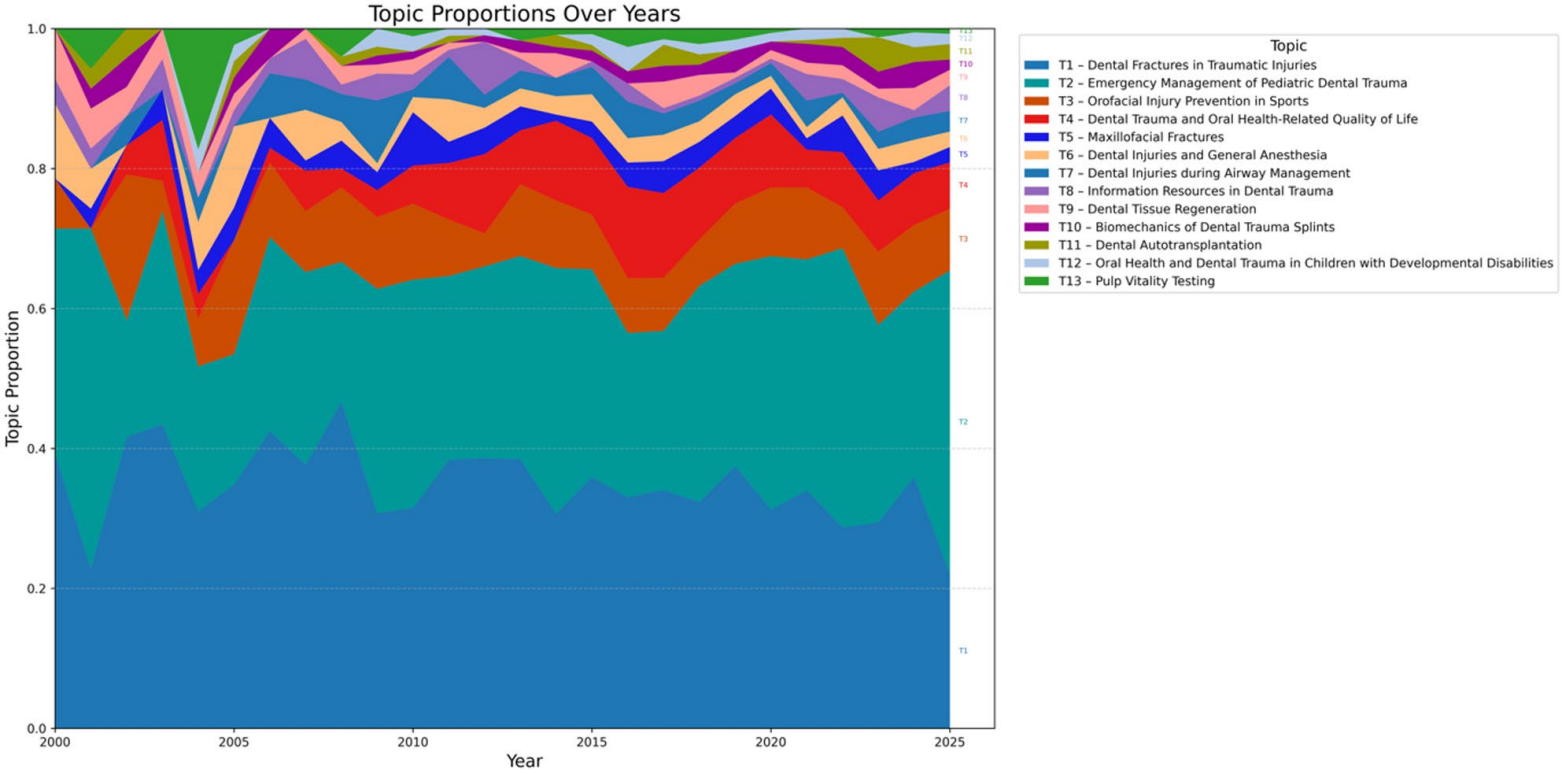
Temporal distribution of topics

The topic distributions in the dental trauma literature between 2000 and 2025 are presented as an area chart in Fig. 4. The chart illustrates the temporal evolution of 13 distinct topics; each color represents a topic, and the area size reflects its relative importance in the literature during the corresponding period.

Over time, it is observed that particularly T1 (Dental Fractures in Traumatic Injuries) and T2 (Emergency Management of Pediatric Dental Trauma) have consistently occupied a substantial portion of the literature. However, in recent years, a marked increase has been noted in T3 (Orofacial Injury Prevention in Sports) and T4 (Dental Trauma and Oral Health-Related Quality of Life).

**Fig. 5:**
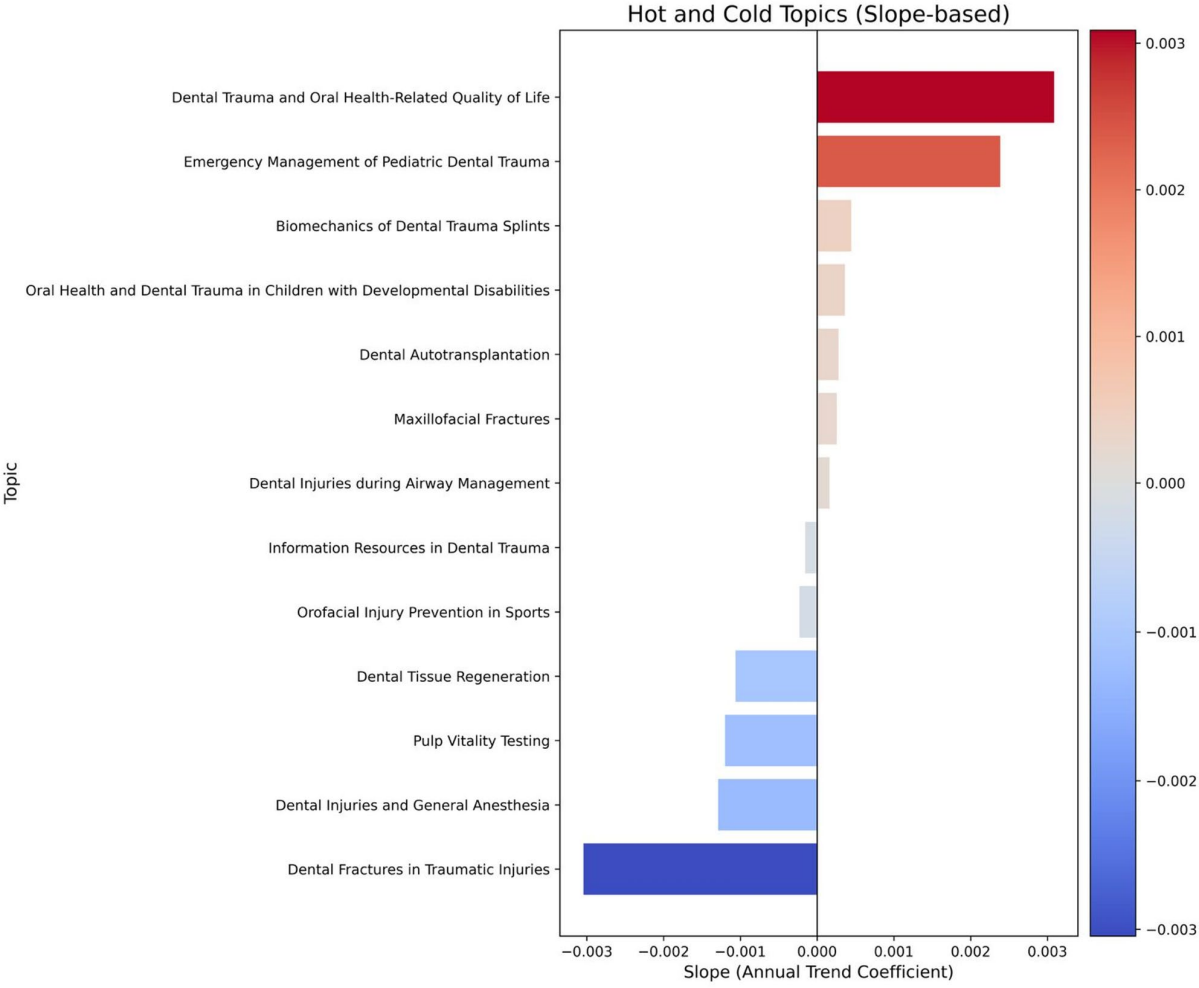
Trends of hot and cold topics

“Dental Trauma and Oral Health-Related Quality of Life” and “Emergency Management of Pediatric Dental Trauma” were identified as hot topics, whereas “Dental Fractures in Traumatic Injuries,” “Dental Injuries and General Anesthesia,” “Pulp Vitality Testing,” and “Dental Tissue Regeneration” were determined as cold topics (Fig. 5).

**Fig. 6:**
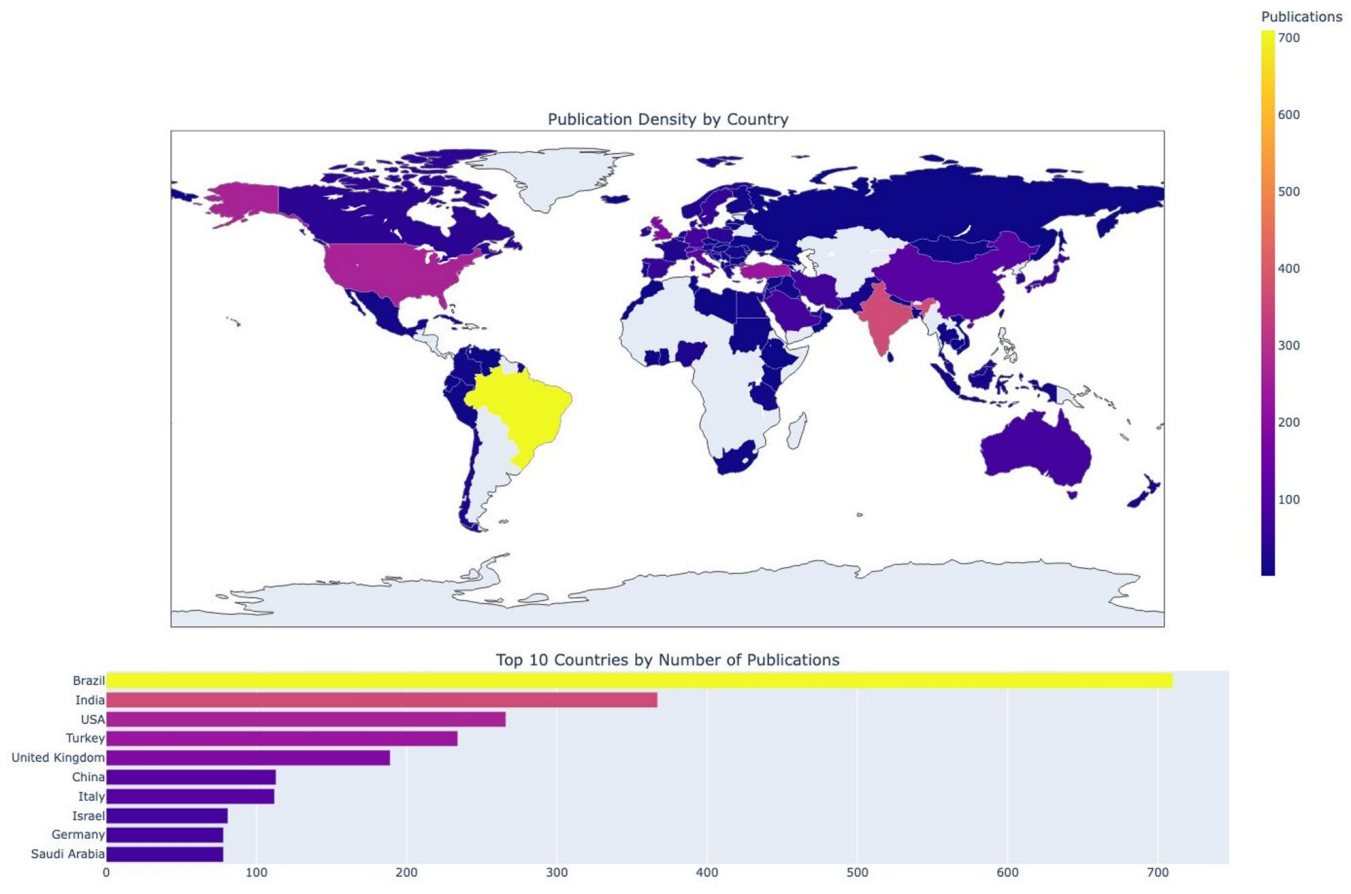
Top 10 most productive countries

Figure 6 presents the top 10 most productive countries in terms of publication output on dental trauma. Brazil stands as the global leader in this field with the highest number of publications, followed by India in second place and the United States in third.

**Fig. 7:**
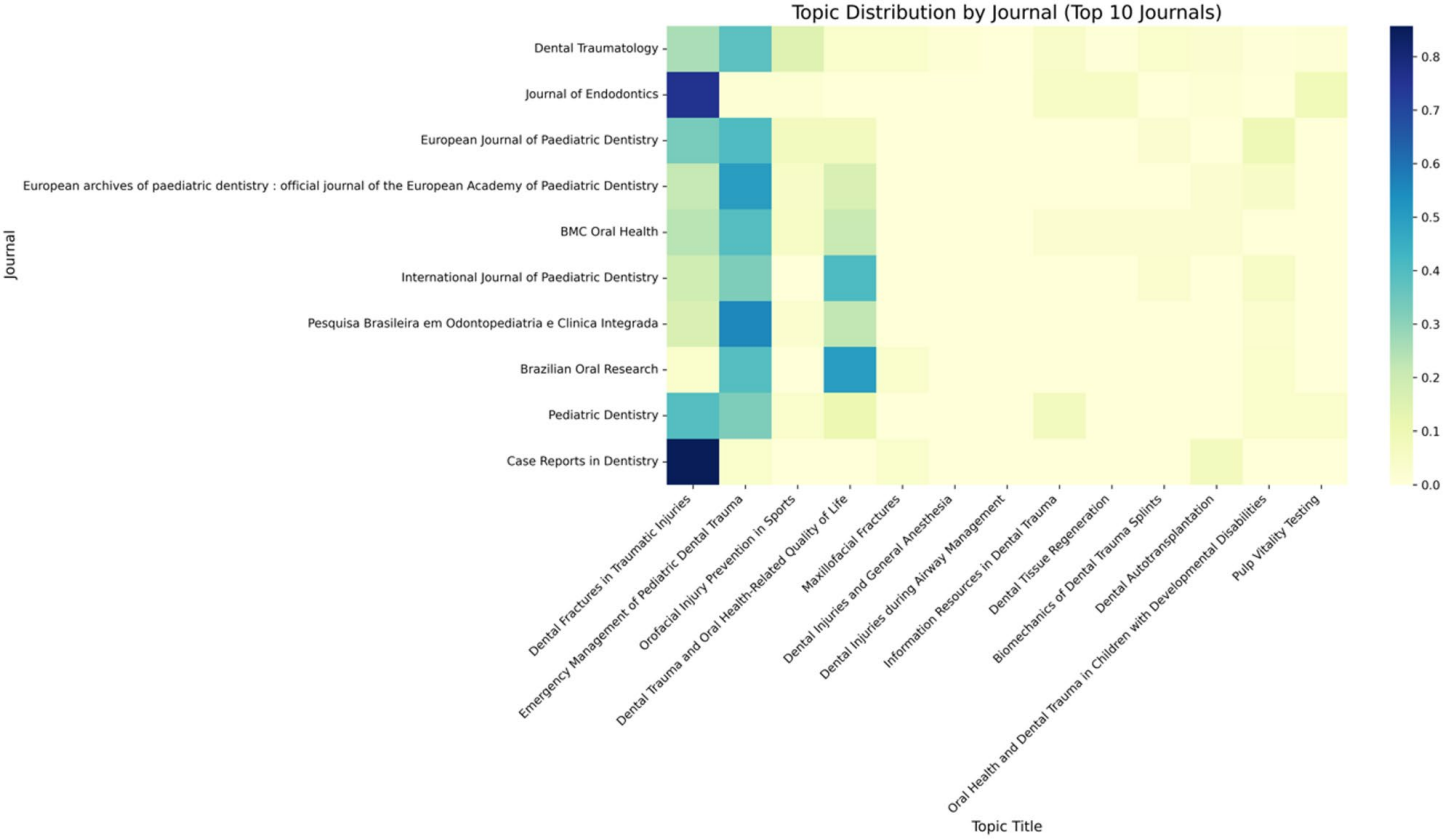
Journal-based topic distribution

Figure 7 displays the topic distribution of the top 10 journals publishing on dental trauma in the form of a heat map. The color intensity reflects the proportion of each journal’s contribution to specific topics. For instance, Dental Traumatology demonstrates a relatively homogeneous topic distribution, whereas the Journal of Endodontics and Case Reports in Dentistry show a marked focus on “Dental Fractures in Traumatic Injuries.”

**Fig. 8:**
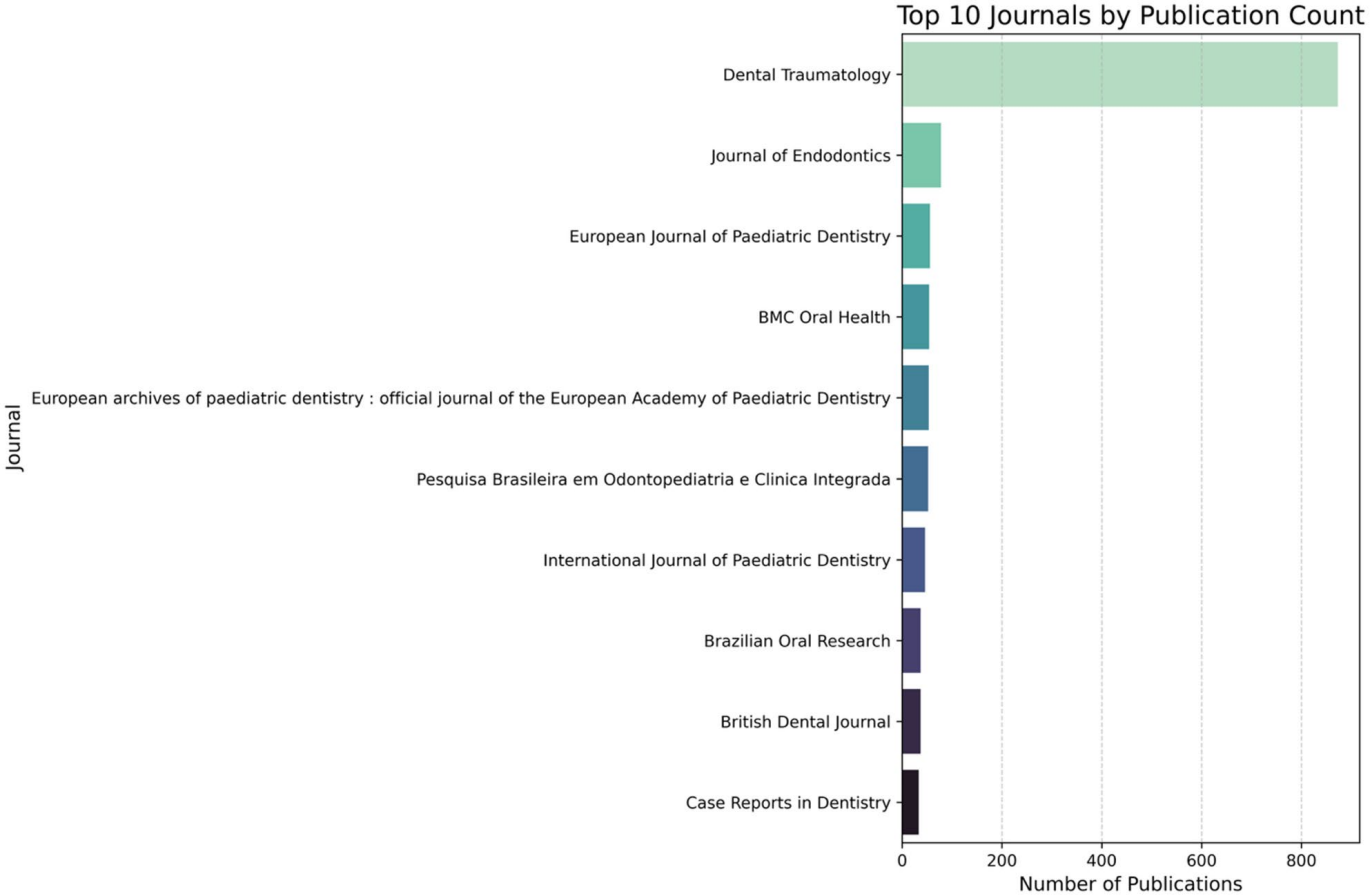
Top 10 most productive journals

Figure 8 shows the top 10 most productive journals by publication volume. Dental Traumatology ranks first with the highest number of publications, followed by the Journal of Endodontics and the European Journal of Paediatric Dentistry.

## Discussion

This study represents the first comprehensive thematic mapping of the dental trauma literature using the BERTopic algorithm. Through this method, the structural themes, temporal trends, and research intensity of the field were identified, and the relationships among countries, journals, and topic clusters were evaluated from a holistic perspective. In this regard, the study provides an original thematic roadmap that makes the shifts in research focus, existing gaps, and potential directions for development in the field of dental trauma visible to clinicians, researchers, and policymakers.

In this study, the Scopus database was utilized as the source of bibliometric data. Scopus is recognized as the most comprehensive abstract and citation search engine for peer-reviewed scientific publications and is therefore frequently employed in literature-based research [23, 24]. Moreover, Scopus provides standardized formats for title, abstract, and keyword data, ensuring high data integrity and reproducibility for NLP-based topic modeling algorithms such as BERTopic [23]. For these reasons, the Scopus database was selected as the most appropriate source to analyze thematic trends in the dental traumatology literature in a reliable, comparable, and systematic manner.

As a result of the BERTopic analysis, the dental trauma literature was found to be divided into 13 themes (Fig. 2). These themes were generated based on the conceptual similarities among the studies in the literature and reflect the structural diversity of the field. The identified topics encompass a wide range of subjects, extending from clinical management to biomechanical and regenerative approaches, and from educational resources to patient-centered outcomes. This indicates that dental trauma has evolved into a multidimensional field of research that integrates diagnosis, treatment, education, psychosocial impact, and technological advancements, rather than being considered merely as a type of injury.

When the topics are ranked according to the number of publications, T1 and T2 exhibit by far the highest volume, whereas topics from T9 to T13 constitute the lowest-volume group, each with fewer than 50 publications (Table 1). The presence of themes with varying publication volumes indicates the thematic diversity within the literature and demonstrates that dental trauma has become an increasingly interdisciplinary field of research intersecting with multiple domains. The themes identified through BERTopic analysis contribute to a holistic understanding of the field by encompassing not only the clinical aspects of dental trauma but also its biological, engineering, and educational dimensions.

The results of our study indicate that the annual number of publications exceeded 50 by 2005, remained above 150 after 2015, and showed a marked increase by surpassing 200 in 2020. This finding demonstrates a remarkable rise in scientific publications in the field of dental trauma over the past two decades (Fig. 3). This upward trajectory is consistent with a previous bibliometric analysis reporting steady global growth in TDI research [8].

The guidelines published by the International Association of Dental Traumatology (IADT) have become the primary references guiding clinical practice. The updated version of the IADT guidelines was published online in May 2020 as four separate articles and quickly became among the most frequently cited sources in the field [25–28]. These guidelines have served not only as essential components of dental trauma education and as guiding resources for clinical practice but also as key references for academic research. An analysis has shown that the 2020 IADT guidelines have been widely utilized by researchers from different disciplines and are among the most frequently cited sources globally [29]. The increase in publications observed after 2020 indicates that this growth is not solely a result of clinical needs but is also significantly influenced by the establishment of international standards, the dissemination of educational resources, and the decisive impact of the IADT guidelines on research directions.

An examination of the temporal distribution of themes in the dental trauma literature reveals the descriptive evolution of research areas over the years and their relative weights in publication volume (Fig. 4). The findings indicate that topics T1 and T2 have maintained the highest publication volumes in the literature for many years and continue to remain at the center of current research. The sustained prominence of T1, characterized predominantly by fracture management and permanent dentition–related treatment decisions, is consistent with previous bibliometric analyses reporting that the majority of publications have historically been treatment-oriented and focused on permanent teeth rather than primary dentition [8]. In contrast, the continuity and upward trajectory of T2 reflect the growing recognition of pediatric dental trauma as a distinct clinical and research priority within the broader field. Taken together, these patterns indicate that clinically oriented domains continue to constitute the structural core of dental trauma research, even as thematic diversification expands around them.

According to our analysis, the T4 theme has shown the strongest positive trend in the literature in recent years, emerging as a hot topic (Fig. 5). This finding indicates that TDI has evolved from being primarily a clinical issue confined to intraoral tissues into a broader public health concern affecting the physical, aesthetic, functional, and psychosocial domains. This trend is consistent with the temporal evolution observed in orofacial trauma research, where increasing attention has been directed toward functional outcomes and disability-related sequelae within a biopsychosocial framework. In a cross-sectional study, temporomandibular joint-related orofacial injuries were shown to frequently result in organic, functional, and situational sequelae, including limitations in mandibular dynamics and social functioning that extend beyond the acute treatment phase [30]. In line with this perspective, our findings indicate that dental trauma, particularly in children, substantially reduces oral health-related quality of life (OHRQoL) through consequences such as functional insufficiency and social isolation [31, 32]. These impacts are not limited to affected children but also extend to their parents, leading to a measurable decline in parental OHRQoL [33]. Collectively, these observations suggest that contemporary dental trauma research increasingly evaluates treatment success not only through clinical outcomes but also through indicators of daily functioning and long-term quality of life [34, 35].

Previous evidence mapping studies in dental traumatology demonstrated that systematic reviews were predominantly concentrated within epidemiologic, therapeutic, and prognostic domains [5, 6]. In addition, the mapping of randomized controlled trials revealed that most randomized controlled trials were clustered within therapeutic subdomains, although their overall number remained limited and methodological quality varied considerably [7]. These domain-based distributions are consistent with the thematic structure identified in the present study. The sustained prominence of T1 corresponds to the strong therapeutic concentration reported in evidence mapping analyses, whereas the increasing visibility of T4 parallels the expanding epidemiologic and prognostic focus within the literature. While previous bibliometric and evidence mapping studies have described the distribution of publications and their methodological characteristics, the present topic modeling analysis provides an additional layer of insight by revealing how research themes have reorganized and evolved over time. This approach allows the identification of structural shifts that may not be fully captured through predefined domain-based classifications.

An analysis of the T1 and T2 themes, which have been among the most dominant topics in the dental trauma literature for many years, revealed that T2 demonstrated a positive trend, ranking as the second hot topic. In contrast, T1 was identified as the theme exhibiting the most pronounced negative trend (cold topic) (Fig. 5).

An examination of the subtopics within the T1 theme revealed that the key concepts forming this cluster were primarily focused on dental fractures in permanent teeth. Crown fractures in maxillary incisors are the most common type of TDI in both dentitions, with a notably higher prevalence in permanent teeth [36, 37]. This trend is reflected in the citation distribution of the 2020 IADT guidelines, where the highest number of citations occurred in the “Fractures and Luxations” and “Avulsion of Permanent Teeth” sections [29]. In contrast, the “Injuries in the Primary Dentition” and “General Introduction” guidelines received fewer citations. These findings indicate that fracture-themed studies have become a relatively saturated area in the literature while maintaining their clinical and scientific significance. In addition, the sub-concepts “root” and “treatment” highlighted within the T1 theme represent the clinical decision-making processes related to the need, timing, and prognosis of endodontic treatment following traumatic dental fractures. A comprehensive review conducted by Abbott (2023) emphasized that the indications and timing of endodontic treatment in injuries such as complicated crown fractures, root fractures, luxations, and avulsions constitute one of the most critical components of trauma management [38]. In this context, the IADT guidelines adopt an individualized approach based on the specific conditions of each case rather than recommending a uniform treatment protocol in the clinical decision-making process. The guidelines emphasize that a particular treatment outcome cannot be guaranteed; however, the likelihood of achieving favorable results can be increased through careful evaluation of clinical judgment, patient characteristics, and treatment options [25]. With the most recent guideline updates, the strengthening of evidence-based clinical practices in trauma management has contributed substantially to resolving many of the fundamental uncertainties in this field. Therefore, the classification of the T1 theme as a cold topic in the literature can be attributed not to a decline in research interest, but rather to the maturation of clinical protocols and the attainment of relative saturation in accumulated knowledge.

The results of our study indicate that dental trauma research has evolved beyond the mere identification and classification of trauma, shifting toward treatment methods, patient education, and quality-of-life assessments. In particular, dental trauma during childhood requires specialized clinical approaches due to immature tooth structures, ongoing root development, behavioral management needs, and differences in prognosis [37]. This situation has increased the focus on pediatric trauma in both clinical practice and academic research, thereby explaining the upward trend observed in the T2 theme.

According to Table 1, the T3 topic has a moderate volume with 230 publications. Based on Fig. 4, no marked upward trend has been detected in this theme; rather, it has maintained a stable position in the literature for many years, showing only limited growth in recent years. It has been reported that sports-related injuries account for one-third of all orofacial traumas, with approximately half of these being TDIs [39]. Dental trauma can occur in both contact and non-contact sports but is more frequently observed in contact sports [40]. Numerous studies have demonstrated that the use of mouthguards significantly reduces the risk of dental trauma [39, 41]. However, it has been reported that mouthguard use among athletes remains insufficient and inconsistent [42]. This situation explains the persistently high incidence of TDIs during sports activities and contributes to the continued and steady presence of this topic in the academic literature over the years.

The T5–T8 topics possess a moderate publication volume and represent themes that broaden the clinical spectrum of dental trauma research. T5 (Maxillofacial Fractures) is associated with high-energy traumas such as traffic accidents, falls, and acts of violence, highlighting the close relationship between dental trauma and maxillofacial injuries. In the literature, the prevalence of dental injuries associated with maxillofacial fractures ranges from 13.1% to 41.8% [43, 44]. This wide range can primarily be attributed to differences in the age distribution of study populations, trauma types, and inclusion criteria. The type and severity of trauma vary with age; high-energy fractures resulting from sports or traffic accidents are more common in younger individuals, whereas the proportion of falls and pathological fractures increases in older adults [43, 45, 46]. Therefore, these variations in the mechanism and localization of trauma lead to substantial differences in the likelihood of developing dental injuries in association with maxillofacial fractures.

T6 (Dental Injuries and General Anesthesia) and T7 (Dental Injuries during Airway Management) focus on iatrogenic dental trauma, particularly injuries occurring during intubation and surgical procedures. In procedures performed under general anesthesia, TDIs represent one of the most frequently reported complications during laryngoscopy or endotracheal intubation. The reported incidence of such injuries varies widely in the literature, ranging from 0.02 to 0.07% in retrospective studies to 3.7–38.6% in prospective studies [47, 48]. This discrepancy can be attributed to the higher sensitivity of case detection in prospective investigations and the underreporting of anesthesia-related dental traumas in certain clinical centers.

The T8 (Information Resources in Dental Trauma) theme focuses on the development of information-sharing tools such as guidelines, online educational materials, and instructional videos accessible to clinicians for trauma management. In recent years, particularly the multilingual availability of the IADT’s web-based content and online guidelines, along with educational videos, has significantly improved access to trauma protocols. These digital resources contribute to clinicians’ ability to develop rapid, reliable, and standardized approaches in diagnosis, treatment planning, and emergency management [49, 50]. Moreover, the global expansion of information accessibility has facilitated the widespread adoption of guideline-based practices in trauma management and has helped reduce heterogeneity in clinical approaches.

Among the topics with relatively lower publication volumes in the dental trauma literature, T9–T13 remain behind the core themes in terms of total number of articles, yet they possess the potential to reflect future research trends in the field. This relative scarcity is not only attributable to the limited attention these areas have received from researchers but also to methodological challenges such as advanced technological requirements, limited sample sizes, the necessity for long-term follow-up, and the complexity of study designs.

In the field of Dental Tissue Regeneration (T9), advances in stem cell and biomaterial research have enabled the biological repair of pulp and dentin tissues following trauma, with a growing number of clinical studies in recent years supporting this potential. The foundation of this trend was established with the publication of the first clinical reports on regenerative endodontic treatment in traumatized teeth in 2008 [51]. However, the relatively low volume of literature on this topic can primarily be attributed to the diversity of clinical protocols, terminological inconsistencies, and the lack of standardization. In the current literature, regenerative endodontic treatments are described using various terms such as “revitalization,” “revascularization,” and “regeneration.” Moreover, significant variations exist regarding irrigation protocols, bleeding induction, scaffold selection, and stem cell sources, which complicate the clustering of studies under a unified thematic axis [52, 53]. Therefore, the limited number of studies within the T9 theme likely reflects the ongoing methodological maturation and standardization process of the field rather than a lack of research interest.

The T10 (Biomechanics of Dental Trauma Splints) theme focuses on the biomechanics of splints used for stabilizing teeth following dental trauma. The rigidity, duration, and material selection of the splint directly influence periodontal ligament healing and the risk of root resorption [54]. A substantial portion of the studies in this area rely on finite element analysis (FEA) to evaluate the stress distribution of various splint materials and designs. In dental traumatology, FEA is a widely used and powerful tool not only for assessing splinting mechanics but also for analyzing the stress patterns generated by traumatic forces in dentoalveolar structures and for evaluating the effectiveness of protective appliances [55]. Nevertheless, this theme is represented by a relatively limited number of publications in the literature. The main reasons include the restricted and heterogeneous nature of clinical cases, the need for highly detailed modeling that incorporates biological dynamics, and the necessity for collaboration between engineering and dental teams [56, 57]. This multidisciplinary approach introduces additional challenges and costs related to logistics, planning, and technical infrastructure.

The T11 (Dental Autotransplantation), T12 (Oral Health and Dental Trauma in Children with Developmental Disabilities), and T13 (Pulp Vitality Testing) themes, which are represented by a relatively small number of studies in the dental trauma literature, have remained limited despite their high potential contributions to clinical practice. Dental autotransplantation, particularly in young patients, is a treatment option that involves the transplantation of the patient’s own tooth to replace a lost one. Owing to continued root development and the preservation of a viable periodontal ligament, this approach offers advantages over implants, such as natural proprioception and maintenance of the alveolar bone [58, 59]. Long-term success rates ranging between 75% and 98% have been reported in appropriately selected cases [58, 60]. However, challenges such as difficulty in identifying suitable donor teeth, variations between donor and recipient sites, the absence of a standardized surgical protocol, and the need for long-term follow-up have limited the publication volume in this area [61].

The T12 theme focuses on a specific patient group prone to trauma due to neurological disorders, cognitive impairments, and behavioral management difficulties. Challenges in accessing this population, communication limitations, socioeconomic factors, and a lack of clinical experience contribute to the low number of studies in this area [62, 63]. Furthermore, insufficient dental awareness among parents or caregivers, difficulties in scheduling appointments, and the limited number of clinics that accept such patients have been reported as additional major barriers [63]. These findings indicate that dental trauma management in individuals with developmental disabilities requires not only clinical approaches, but also multidimensional strategies aimed at enhancing caregiver-based awareness and improving access to dental care services.

Pulp vitality tests aim to assess the viability, sensitivity, or responsiveness of teeth following trauma using non-invasive methods. Within this context, techniques such as laser doppler flowmetry, pulse oximetry, thermal tests, and electric pulp tests are utilized [64]. Our study revealed that the number of publications addressing the use of these diagnostic tests in the literature remains limited. This may be attributed to the restricted reliability, accuracy, and reproducibility of the currently available tests [65]. Further scientific research is needed to overcome these limitations; however, the current literature has not yet adequately met this need.

According to our study, the distribution of publications across countries in the dental trauma literature shows that Brazil is the clear leader, with more than 700 publications, followed by India and the United States (Fig. 6). This pattern may be associated with both the higher clinical incidence of TDIs and strong academic productivity of research institutions in this field. Türkiye and the United Kingdom stand out as leading countries within the European region (Fig. 6). Additionally, the inclusion of China, Italy, Israel, Germany, and Saudi Arabia among the top ten countries indicates that dental trauma research is not confined to nations of a particular economic status; rather, it reflects the influence of multifactorial determinants such as sociocultural context, dental education systems, health policies, research infrastructure, and access to healthcare services.

Artificial intelligence (AI) is rapidly expanding its applications in dentistry, particularly in areas such as image analysis, diagnosis, and treatment planning [66]. However, the BERTopic modeling performed in our study revealed that in the dental trauma literature, AI-related keywords such as “artificial intelligence,” “deep learning,” “convolutional neural network,” “decision tree,” “support vector machines,” “generative adversarial networks,” and “machine learning” did not form a distinct thematic cluster. Moreover, no subtopics associated with these concepts were identified. Similarly, bibliometric analyses focusing on the applications of AI in dentistry have not reported dental traumatology as a prominent research domain [67, 68]. This finding indicates that AI-based studies in dental traumatology are still in an early and limited developmental stage compared to the rapid advancements observed in other dental disciplines. It also underscores the need for future research to develop innovative AI-driven approaches aimed at enhancing diagnostic accuracy and standardizing treatment decision-making processes.

According to our study, the journal Dental Traumatology was identified as the most dominant source in terms of both publication volume and thematic diversity (Figs. 7 and 8). However, the journal did not focus on a single topic; rather, it covered a broad range of dental trauma subjects in a relatively balanced manner. This indicates that the journal functions as a multidisciplinary and integrative platform representing the overall scope of dental traumatology. The Journal of Endodontics and Case Reports in Dentistry, on the other hand, were found to concentrate particularly on the theme of “Dental Fractures in Traumatic Injuries” (Fig. 7). This trend may be associated with the high frequency of traumatic cases involving endodontic complications and root canal treatment requirements. In terms of total publication count, Dental Traumatology was followed by the Journal of Endodontics, European Journal of Paediatric Dentistry, and BMC Oral Health. This distribution suggests that dental trauma research extends beyond a single specialized journal and is also represented across multidisciplinary journals in pediatric dentistry, endodontics, and general dentistry. Overall, this pattern reflects the inherently interdisciplinary nature of dental trauma and suggests that the trend toward cross-specialty publication is likely to continue in the future.

This study has certain methodological and scope-related limitations. First, it includes only articles indexed in the Scopus database and published in English; therefore, studies published in other languages or indexed in different databases were excluded. Second, each topic cluster generated by the BERTopic algorithm was labeled with meaningful titles based on distinctive keywords and subsequently interpreted by expert researchers. Although this labeling process was based on consensus among three independent researchers, the assignment of topic names inevitably involves a degree of subjectivity. Third, in our analysis, institutional contributions were evaluated solely based on the affiliation of the first author, which may not fully reflect the overall institutional distribution of multicenter or inter-institutional studies. Fourth, since BERTopic modeling relies on vector representations derived from titles, abstracts, and keywords, thematic details present in full texts but not explicitly stated in abstracts were not included in the analysis.

Future studies may contribute to a broader evaluation of the dental trauma literature. In particular, multi-source analyses that include studies published in different languages and indexed across multiple databases would allow a more comprehensive mapping of the existing thematic distribution.

## Conclusion

This study presents a large-scale thematic mapping of the dental trauma literature using the BERTopic algorithm, revealing the structural and conceptual evolution of the field over the past two decades. The analysis identified thirteen distinct themes reflecting the multifactorial and multidisciplinary nature of dental trauma. The findings indicate that the field has long been shaped by clinically oriented topics, particularly around traumatic tooth fractures and the emergency management of childhood dental injuries.

However, in recent years, the growing emphasis on quality of life, psychosocial impacts, and patient-centered assessments demonstrates that dental traumatology is evolving beyond a purely biomedical domain to encompass behavioral and societal dimensions. Moreover, emerging but relatively limited research areas such as tissue regeneration, biomechanical analysis, autotransplantation, and trauma management in individuals with special needs represent innovative themes that may expand the scientific boundaries of the field in the future. Dental trauma research is increasingly assuming a distinctly multidisciplinary character. The interaction between clinical protocols, biomechanical analyses, and biotechnological applications is broadening the scope of therapeutic approaches. Although still underrepresented, AI-based systems are expected to become an integral component of this multidisciplinary framework in the coming years.

## Data Availability

The dataset used for the BERTopic analysis, including the processed text corpus, topic modeling inputs, and all derived topic outputs, can be shared without restriction. Although the raw records were originally retrieved from the Scopus database, the authors can provide the dataset and all associated analysis scripts upon reasonable request.

## Abbreviations

AI: Artificial Intelligence
c-TF–IDF: Class-based Term Frequency–Inverse Document Frequency
FEA: Finite Element Analysis
HDBSCAN: Hierarchical Density-Based Spatial Clustering of Applications with Noise
IADT: International Association of Dental Traumatology
NLP: Natural Language Processing
NLTK: Natural Language Toolkit
OLS: Ordinary Least Squares
OHRQoL: Oral Health-Related Quality of Life
TDI: Traumatic Dental Injury
UMAP: Uniform Manifold Approximation and Projection
